# Antenatal education for childbirth: Labour and birth

**DOI:** 10.18332/ejm/120002

**Published:** 2020-04-23

**Authors:** Lisa Cutajar, Michelle Miu, Julie-Anne Fleet, Allan M. Cyna, Mary Steen

**Affiliations:** 1 UniSA Clinical and Health Sciences, University of South Australia, Adelaide, Australia; 2 Department of Women’s and Children’s Health, Birth Unit, Nepean Hospital, Penrith, Australia; 3 Anaesthesia and Pain Management, Nepean Hospital, Penrith, Australia; 4 Acute Care Medicine, University of Adelaide, Adelaide, Australia

**Keywords:** positive suggestions, antenatal education, labour and birth, childbirth

## Abstract

**INTRODUCTION:**

This study aimed to identify the way information is described and presented by childbirth educators during antenatal classes for expectant parents, and analyse the language structures used when discussing labour and birth.

**METHODS:**

This cross-sectional study of antenatal education was conducted at a single tertiary referral centre for Maternity Care in Western Sydney, Australia. All childbirth educators (n=3) were recorded whilst providing information to parents during antenatal classes. Audio data were subsequently transcribed and then analysed by two researchers, independently categorising the various language structures and types of information provided. This is the second study in a series of antenatal education topics.

**RESULTS:**

During the labour and birth class, information statements were the predominant language structure that was spoken with 241 of 655 statements; negative statements were the next most frequent at 119 while there were 79 positive statements. The second stage of labour had a greater proportion of negative statements for two educators, followed by information and positive statements combined. Misinformation statements were minimal for this topic however, and there was an absence of any statements discussing the rest period between contractions.

**CONCLUSIONS:**

The findings further emphasise the need to examine the language used by health professionals when educating parents. Negative statements during antenatal education are still common despite research in other contexts suggesting that these are potentially unhelpful. Further research into the language and suggestions used during antenatal education is required to determine whether improved outcomes seen in other contexts are confirmed in the childbirth setting.

## INTRODUCTION

This study aimed to identify the way information is described and presented by childbirth educators during antenatal classes for expectant parents, and analyse the language structures used when discussing labour and birth, in particular, the use of storytelling metaphors, listening and acceptance, utilisation, reframing, positive and negative suggestions.

Childbirth is one of the most significant events in a parent’s life and has the potential to be an exhilarating and fulfilling experience for some or a frightening anxiety provoking experience for others^1,2^. Structured antenatal classes have developed worldwide as traditional methods of information sharing have declined and expectant parents look for strategies to prepare for childbirth and parenthood^3,4^. Dissemination of antenatal information is a dynamic process, constantly evolving to meet the needs and expectations of women and their partners. Current research and guidelines recommend that health professionals examine the language they use when providing care^5-7^. The sentiment of ‘watching what you are saying’ was identified by Robertson^8^ as an important tool for professional development and accountability. Twenty years later, the need for health professionals in the maternity setting to consider their use of language continues to be highlighted. The question of how language may impact on a woman’s self-efficacy in labour and birth was examined by Campbell and Nolan^9^ using a grounded theory approach. During that study the researchers specifically examined the aims, language and actions of yoga for pregnancy teachers. The study identified four themes: building confidence, creating a sisterhood, modelling labour, and enhancing learning; and each of these themes had various subthemes. Of the four themes, building confidence involved the use of positive language, imagery and positive affirmations, to ‘emphasise how strong and capable women’s bodies were and how beautiful birth can be’^9^. Creating a sisterhood incorporated storytelling from other experienced mothers in the group and the childbirth educators. While enhancing learning included creating an atmosphere through tone of voice, soothing words, and use of metaphor. Modelling labour was another theme and focused on the repetition of words and postures in order to imbed phrases in the woman’s mind^9^. The language of encouragement was also discussed by Leap and Hunter^10^ as a way of stimulating positive motivation, with antenatal groups being identified as one opportunity where this can occur. This is particularly important because positive language has been linked to improved outcomes^7^.

The ‘Clinical Practice Guidelines in Pregnancy Care’, of the Department of Health^11^, outlines the aims of structured childbirth education. These aims direct content of antenatal childbirth classes and involve planning for childbirth, which include preparing women and their partners for the pain of labour, to build confidence in their ability to labour, and give birth without pain relief^11^. Internationally, there is a move to incorporate respectful collaborative communication with a growing consensus to emphasise the importance of examining what we say and how we say it, to be aware of actual words used, and their tone and demeanor^6^. The World Health Organisation (WHO) suggests responding with a positive attitude to woman’s needs, preferences and questions during the course of structured antenatal classes, with particular attention on providing information in a clear and concise manner^5^.

In this single-centre cross-sectional study the aim was to identify the way labour and birth information is presented during antenatal classes by childbirth educators and assess consistency of content and language structures.

## METHODS

### Study design

This cross-sectional study observed childbirth educators as they presented a course of antenatal classes. Detailed methodology has been reported previously^12^. In summary, this study utilises directed content analysis with predetermined codes. The study was approved by local regional ethics committee (HREC Study 16/45 – LNR/16/NEPEAN/74) and University of South Australia (Application ID: 202481). Written informed consent from both educators and parents was obtained. Recruitment of personnel attending antenatal classes at a tertiary referral centre for maternity care in NSW, Australia, occurred between February and April 2017.

### Data collection

Informed consent to record antenatal classes was obtained from all three childbirth educators, enrolled couples and student midwives attending the class. The educators were fitted with a lapel microphone and antenatal classes were recorded in their entirety. Data were collected from each of the childbirth educators relating to educator qualifications and experience^12^ by means of a standardised questionnaire as well as information disclosed to parents during sessions.

### Data analysis

Direct content analysis using predetermined codes based on an observational study by Slater et al.^13^ were used. The predetermined categories^13^ were an extension of those used in similar communication studies by nurses^14^ and radiologists^15^, examining the use of positive and negative statements and suggestions. Data that could not be coded initially were analysed later to determine if they represented a new category or a subcategory of existing coding categories^16^. Categories used included: storytelling, imagery, metaphor, information statements, direct and indirect commands, and positive and negative suggestions^13^ (Table 1). Transcripts were generated from audio recordings and subsequently analysed by two researchers independently to ensure reliability of the analysis^17^. The categorised language structures were compared to assess consistency of analysis^13^. When concordance was not observed the discrepancies were discussed and a third researcher was consulted. Data are presented as codes, and examples of statements are provided. Descriptive statistics were used to identify frequency and potential trends.

**Table 1:** Language techniques with definitions and examples

*Language technique*	*Definition*	*Example*
Positive suggestions	Implies a positive experience or therapeutic outcome.	‘The baby's head is going to borrow that space on the way down and that’s absolutely fine.’ ‘Contractions bring you closer to your baby.’ ‘But being active, it's efficient.’
Negative statements	Implies a negative experience or outcome.	‘Crowning and burning, right there is burning and stinging.’ ‘You could easily mistake that for tearing.’ ‘So, she will say she will feel like the baby is coming through the bowel.’
Information statements	Neutral statements providing information concerning the procedure.	‘So generally, they are 5 minutely and they are lasting 40 seconds or longer, and they are making you concentrate.’ ‘Transition is that time between the first and the second stage, and the cervix is nearly fully dilated.’
Story telling	A means of using imagery and focusing attention.	‘And I haven't seen it for a long time but as fresh as last night guess what, out in one or two pushes – this little boy was here!’
Checking in	Statements enquiring about the groups’ understanding of information.	‘So, tell me what is on your mind? What would you be thinking that you would want to do?’
Metaphor	A phrase used to convey meaning in other then the literal sense.	‘Dads and support partners, you are the Oxytocin warriors, you are getting the place calm. You are keepers of the birth place.’
Direct commands	Specific instructions.	‘If there is anything you are worried about, that's when you ring the birth unit.’
Indirect suggestions	Often prefaced with ‘Most women …’and implies a similar experience.	‘So, if contractions are regular and last more than 30 seconds and are closer than 5 min apart usually most people are ringing up.’

## RESULTS

All childbirth educators (n=3) consented to participate and are de-identified as M1, M2 and M3. Class sizes ranged from seven to eleven couples. All, but one woman, were primipara and all women were over 25 weeks’ gestation at the commencement of classes.

**Figure1:**
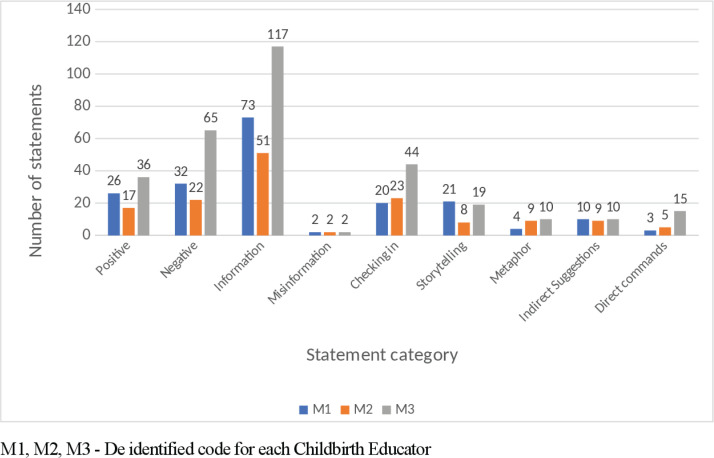
Comparison of language techniques used when discussing labour and birth

Time spent discussing labour and birth was 40 minutes for two childbirth educators (M1 and M2) and 60 minutes for M3. The number of statements varied with M1 = 191, M2 = 146, and M3 = 318 statements. All three childbirth educators used information statements frequently. Negative statements were common whilst ‘checking in’ and positive statements were identified and observed to be similar in frequency (Figure 1). Language structure examples are provided in Table 1. Metaphors were more often used to describe or explain physical changes that occurred during labour. For example, all three educators used a ‘balloon’ description, as either a metaphor for the uterus, e.g. ‘the uterus … is a balloon shaped muscle’ (n=2), and the third educator referred to the amniotic sac as a balloon, e.g. ‘so this baby is in a bag of fluid and the bag is like a balloon and that is what the membranes are’.

**Figure 2:**
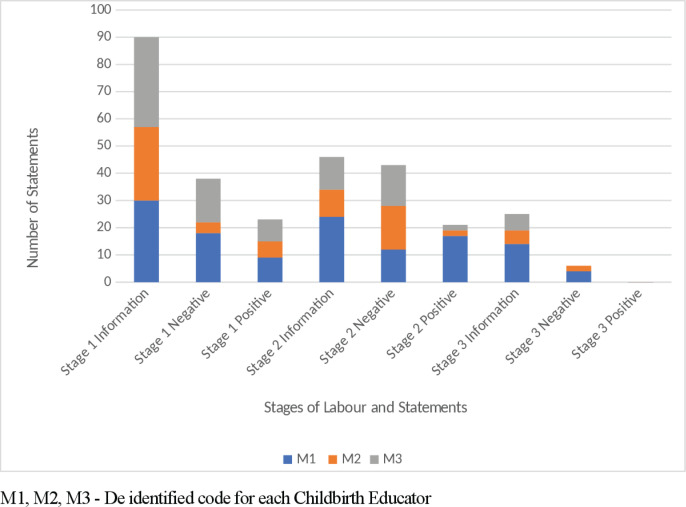
Comparison of information, positive and negative statements made by childbirth educators when discussing stages of labour

**Figure 3:**
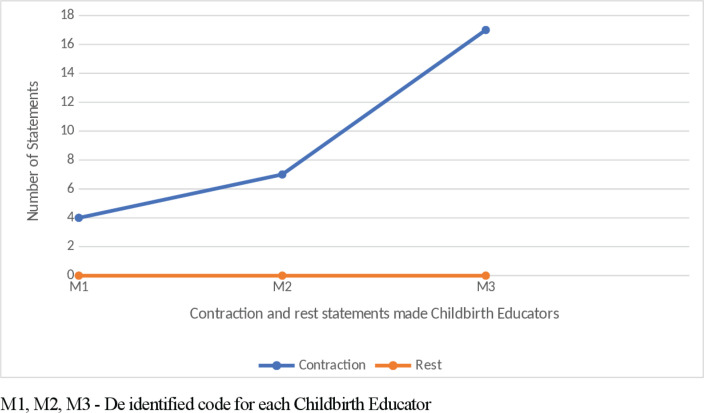
Comparison of labour contraction statements and labour rest period statements

Misinformation was a new category identified and observed^12^, with all three childbirth educators having a total of two episodes each (Figure 1). Examples of misinformation included the description of contractions, e.g. ‘So they come up onto the tummy, in out, up down, all around, what happens?’, and the explanation of the stages of labour, e.g. ‘the placenta is left in there and that is the beginning of the second stage’. Frequency of positive and negative suggestions expressed by childbirth educators when stages of labour were discussed are shown in Figure 2. Information statements were the primary language technique conveyed by the childbirth educators for each of the stages of labour. Examples of stage one early-labour information statements included: ‘so there is a longish cervix and in early labour it has some shortening to do in that period of time’; ‘sometimes they are not painful, sometimes they are just pressure, sometimes they are just tightening’; and ‘the beginning of labour is intense regular contractions’. Active-labour information statements included: ‘these contractions are going to be coming eventually every 2–3 minutes’ and ‘on average it is around twelve hours’. Transition was explained as: ‘transition is that time between the first and second stage and the cervix is nearly fully dilated’ and ‘so contractions work to dilate the cervix’. Analysis of the frequency of word descriptors voiced when explaining contractions and rest periods between contractions are shown in Figure 3; no mention of the rest period between contractions was made by the childbirth educators. Information statements and positive suggestions are combined in Table 2 to demonstrate differences between childbirth educators for frequency of negative suggestions expressed when each stage of labour was discussed. Stage one and stage three have greater positive and information statements (Table 2). When discussing stage two, more negative suggestions were expressed by M2 and M3 than positive and information statements combined. Examples of negative statements made when discussing stage two included: ‘there is the burning and stinging’ and ‘the vagina will stretch, stretch, stretch, but you could easily mistake it for tearing’.

**Table 2:** Comparison of combined positive and information statements with negative statements when discussing the stages of labour and birth

	*Positive and information statements n (%)*	*Negative statements n (%)*	*Total number of statements*
M1 Stage 1	39 (68)	18 (32)	57
M2 Stage 1	33 (89)	4 (11)	37
M3 Stage 1	41 (72)	16 (28)	57
M1 Stage 2	41 (77)	12 (23)	53
M2 Stage 2	12 (43)	16 (57)	28
M3 Stage 2	14 (48)	15 (52)	29
M1 Stage 3	14 (78)	4 (22)	18
M2 Stage 3	5 (71)	2 (29)	7
M3 Stage 3	6 (100)	0	6

M1, M2, M3 – De-identified code for each childbirth educator.

**Figure 4:**
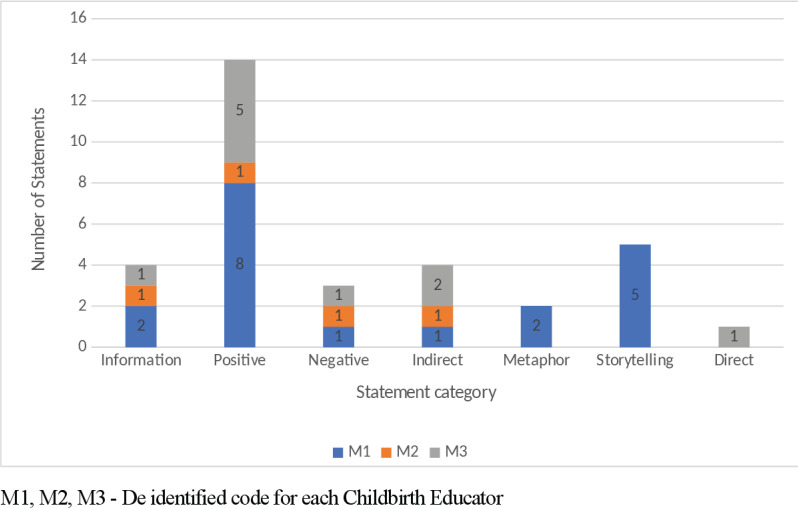
Statements made by childbirth educators when discussing support in childbirth

Frequency of the language structures used when the role of a support person in labour was discussed are shown in Figure 4. The support person was identified as either a partner or significant other, not as a midwife or health professional. Positive suggestions are demonstrated as the dominant technique, followed by storytelling and information statements (Figure 4).

## DISCUSSION

This study used audio recordings of childbirth educators to analyse how labour and childbirth were explained to women and their partners, during childbirth classes. It is a separate topic paralleling the design and goals of a previously published study^12^.

Childbirth educators aim to provide ‘accurate, realistic, practical information’ to guide the women and their partners on the pregnancy, birthing and parenting journey. Guidance from the Department of Health^11^ not only focuses on the giving of information but also specifies ‘preparing women and their partners for childbirth, including building woman’s confidence in their ability to labour and give birth’. The building of confidence can be linked to physicality of the environment and language with which the information is presented. The sizes of classes ranged from seven to eleven couples. The optimal group size of twelve to twenty adults, which best facilitates discussion and interactions within a group, has been recommended by Nolan^18^. Small classes were identified by the Department of Health^11^ as a factor that parents prefer because it encourages social networking and is less intimidating than large classes, thus making it easier for women and partner/support person to ask questions.

Antenatal education of labour and birth included the stages of labour, contractions and role of the support person. Information statements were the dominant language technique used by all childbirth educators, followed by negative suggestions. ‘Checking in’ was more frequent then positive suggestions, while misinformation was far less common in this study compared to the labour epidural topic^12^. Misinformation that did occur involved confusing descriptions about contractions and the stages and phases of labour. Possible reasoning for increased ‘checking in’ and decreased misinformation could be that childbirth educators have a greater expertise with this subject matter and are more at ease with addressing any queries via the ‘checking in’ process, as opposed to when discussing the labour epidural.

The three stages of labour and contractions were discussed in depth by each childbirth educator. Stage one received most attention, as reflected by the number of information statements. This was due to early labour, established labour, waters breaking and contractions all being discussed within the first stage. Each of the educators utilised definitions for early labour and established labour with information focused on instructing women and their partners to recognise the stages and remain in the home environment until labour established. This is consistent with findings of Ferguson et al.^19^ who concluded that one of the positive effects of antenatal education was less false labour admissions.

Contractions and how they differ during stages one, two and three are explained in terms of pain, frequency, intensity, and duration. However, there was an absence of discussion pertaining to the rest period between contractions by all childbirth educators. The ‘Midwifery wave’ concept, as described by Leap and Hunter^10^, is used to explain that the contraction starts slowly, builds to a peak, lasts about a minute before it dies down and is followed by a rest. The omission of this rest denies birthing mothers of the opportunity to refocus, relax, feel more confident and mentally prepare for the next contraction^10^. Inclusion of the rest-contraction-rest cycle, as opposed to only discussing contractions, can function as an information statement that possibly has the power to prime a mother for positive behaviour and perceptions. This was identified by Campbell and Nolan^9^ when they discussed the concept of ‘building confidence’, which involved the use of positive language, imagery and positive affirmations to ‘emphasise how strong and capable the woman’s bodies were and how beautiful birth can be’.

During the discussion of stage two, from full dilatation to the birth of baby, it was identified that negative statements made by two childbirth educators were greater than both information and positive statements combined. Studies have identified that the use of positive and negative suggestions may have implications for birth expectations and experiences of women and their birth partner^20^. Suggestions are a communication technique that can lead to subconscious non-volitional changes in perception, mood, and behaviour^21^. Negative statements were in reference to the perceptions of pushing and birth, with examples such as: ‘I can feel stinging and burning sensations when I am pushing now’; ‘But you could easily mistake it for tearing’; and ‘So that is right crowning and burning, right there is the burning and stinging’. These warnings and references to painful and unpleasant sensations may provoke a nocebo effect^15^. The focus on negative suggestions has potential to shape and influence the experience of women during their labour and birth, particularly since suggestibility increases when people are highly anxious, distressed, in pain, and during pregnancy^22^. Hollander et al.^23^ identified factors that impacted on the incidence of post-traumatic stress disorder experienced by women following childbirth. These factors included lack of communication and the provision of information during the antenatal period. Women stated that their traumatic birth experience could have been prevented had their caregivers communicated or explained more or listened more. Hollander et al.^23^ identified that it was the ‘interactions rather than interventions’ that resulted in the trauma. Childbirth educators, therefore, have a potentially crucial role in providing women and their support people with neutral information and skills to navigate the birth experience.

Support in labour was discussed by each of the educators and all referred to the partner, not the midwife or the primary health professional. Hodnett et al.^24^ found that ‘women who received continuous labour support were more likely to give birth spontaneously’ and they ‘were less likely to use pain medications, were more likely to be satisfied’ regardless of who provided the continuous care. Leap and Hunter^10^ also outlined evidence that shows simply having someone in the room, ‘being there’, is enough to shorten the labour. According to Ferguson et al.^19^ one of the effects of antenatal education is an increase in partner involvement in the birth process. As a result, it is crucial to consider what is being said to empower partners^25^. When discussing support of partners in labour, positive suggestions were the primary language technique used by all three educators. Examples, provided in Table 1, of positive suggestions used when referring to support people included: ‘they need to bring that confidence, that “can-do” attitude’; and ‘dads and support partners you are the oxytocin warriors, you are the keepers of the birth space!’. The inclusion of positive suggestion is important as parents will form their expectations based on how antenatal information is communicated, which may be a key determinant of their subsequent experience^23,26^.

Discussion within the class was stimulated via ‘checking in’, as well as stories told by each of the educators. Carolan^27^ identified that the value of storytelling is widely recognized from a sociological point of view, particularly when interpreting life events. Savage^28^ explains birth stories shared by woman as the passing of wisdom ‘from one who knows’ to those ‘that need to know’. This was evident in an unusual occurrence in M3’s group where one woman was expecting her third child. The woman shared her birth stories, which were incorporated into the information provided by the childbirth educator. In particular, the woman’s story about a ‘posterior birth’ enhanced and provided perspective for the class regarding the educator’s information about positioning in pregnancy.

According to Kay et al.^29^, ‘articulating the birth experience gives it structure and once the experience has structure there is the potential for meaning to be determined and emotional responses considered’. A birth story described by M1 identified the relationship between adrenaline and oxytocin. The birth story gave meaning to a physiological process and expressed that woman laboured when they were relaxed and unobserved. Kay et al.^29^ also identified that when telling positive birth stories women hear of the strength and power in birthing and may be assured of their capacity to birth physiologically. This was evident in the following examples from M1: ‘I remember the first time the woman in the unit gave birth in the “all fours” position, we talked about it for weeks!’, and M2: ‘I haven't seen it for a long time but as fresh as last night guess what, out in one or two pushes – this little boy was here!’. Conversely, women that encounter negative birth stories may associate birth with suffering, risk, and fear^29^. Within the topic of labour and birth there was an absence of negative storytelling.

Metaphor is defined as a phrase used to convey a meaning other than the literal sense. When categorising the language structures within this study, similes were included within the category ‘metaphor’ (Table 1) as they are understood in a similar manner^30^. Metaphor usage was greater during the labour and birth session when compared to the labour epidural discussion^12^. In the previous study when childbirth educators discussed labour epidural, the metaphors used were neutral, purposeful and provided direction by describing an action^12^. For example, ‘so ultimately the small of your back is curving out like a C’ was used to promote the optimal positioning for an epidural procedure^12^. According to Littlemore and Turner^31^, metaphor allows for exploration of new experiences by relating it to something that is familiar, tangible or common. Metaphors that were used with labour and birth were largely comparative and served the purpose of linking the abstract to the known^32^. These metaphors became a shared common language from which discussion was stimulated due to the vivid descriptions that were provided. Examples include ‘the uterus is a balloon shaped muscle’ and ‘…. the bag is like a balloon and that is what the membranes are’. Other metaphors were used to describe the changing consistency of the cervix ‘from a hard (knocks on the whiteboard) to like a soft silicon’. There were instances where metaphors had the potential to influence negative perceptions such as the crowning of the baby was referred to as ‘the ring of fire’. By examining the metaphors used within labour and birth education it is evident that they have potential to be used as information and positive or negative suggestions. Leap and Hunter^10^ cautioned the use of metaphors due to potentially different impacts on the woman. For instance, the use of the term ‘waves’ to describe contractions may be a positive suggestion for a woman fond of the sea surf, but has also the potential to invoke fear in a woman who is scared of being overwhelmed by the sea.

### Implications for practice

Current research and changing guidelines are directing and recommending that health professionals examine the language utilised when providing care. This study demonstrates that this could be extended to include the information provided, particularly when discussing the physiological truisms of contraction and rest periods. This is especially important when it is acknowledged that positive communication has been directly linked to improved outcomes.

The present research will have important implications for childbirth education in the future to enhance the language educators and health professionals use when discussing the labour and birth process. In particular, it will serve to identify attributes of language that can be utilised to enhance the childbirth experience by suggesting a sense of control and empowerment rather than fear, distress, and pain. Future research focusing on the perceptions of first-time expectant parents who have attended antenatal classes, both before and after the birth experience, would further inform how the language used influenced their experience.

### Strengths and limitations

The strengths of the study included 100% participation of childbirth educators from a single tertiary institution, and the collection of greater than 700 statements and data elements.

A limitation of the study was that only a single recording of each specific class was conducted, which potentially missed variance in individual practices. Further, the presence of recording equipment may have influenced what was presented, although previous observational studies have not reported this^33^. The researchers also acknowledge that the words used by the childbirth educators form only one part of the communication strategy. The timing, tone and pace of certain words may also impact on how a concept is perceived and should be explored in future research.

## CONCLUSIONS

The findings of this study suggest a need to provide more consistent evidence-based antenatal information. Negative statements during antenatal education are still common despite research in other contexts suggesting these are potentially unhelpful. Positive language, motivation and the provision of meaningful accurate information in the antenatal education setting and the effects of such information on childbirth outcomes are likely to be fruitful areas for further research.
